# Folic Acid Conjugated δ-Valerolactone-Poly(ethylene glycol) Based Triblock Copolymer as a Promising Carrier for Targeted Doxorubicin Delivery

**DOI:** 10.1371/journal.pone.0070697

**Published:** 2013-08-21

**Authors:** Lekha Nair K, Sankar Jagadeeshan, Asha Nair S, G. S. Vinod Kumar

**Affiliations:** 1 Chemical Biology, Rajiv Gandhi Centre for Biotechnology, Poojappura, Kerala, India; 2 Cancer Research, Rajiv Gandhi Centre for Biotechnology, Poojappura, Kerala, India; King's College London, United Kingdom

## Abstract

The aim of this study is to test the hypothesis that the newly synthesized poly(δ-valerolactone)/poly(ethylene glycol)/poly(δ-valerolactone) (VEV) copolymer grafted with folic acid would impart targetability and further enhance the anti-tumor efficacy of doxorubicin (DOX). Here, folic acid conjugated VEV (VEV-FOL) was synthesized by a modified esterification method and characterized using IR and NMR. DOX loaded VEV-FOL micelles were synthesized using a novel solvent evaporation method and were obtained with a mean diameter of 97 nm with high encapsulation efficiency and sustained *in vitro* release profile. Comparative studies of polymer micelles with and without folate for cellular uptake and cytotoxicity were done on folate receptor-positive breast cancer cell line, MDAMB231. The intracellular uptake tests showed significant increase in folate micellar uptake when compared to non-folate-mediated micelles. MTT assay followed by apoptosis assays clearly indicated that folate decorated micelles showed significantly better cytotoxicity (IC_50_ = 0.014 µM) and efficiency to induce apoptosis than other treated groups. Moreover, a significant G2/M arrest was induced by DOX loaded VEV-FOL micelles at a concentration where free drug failed to show any activity. Thus, our results show that the folic acid-labeled VEV copolymer is a promising biomaterial with controlled and sustainable tumor targeting ability for anticancer drugs which can open new frontiers in the area of targeted chemotherapy.

## Introduction

Advances in nanotechnology, including ours, have analyzed a variety of nanoscaled carriers for controlled drug delivery for a variety of ailments [1]–[3]. However, in chemotherapy a significant issue determining its effectiveness is the ability of drug carriers to control the location and time over which drug release occurs. This challenge has motivated the development of nanoparticle systems that are designed to release their drug load at the target site in a controlled manner. Non-specific delivery of anticancer agents often results in damage of healthy organs. Many of the chemotherapeutic treatments available are accompanied by such serious side effects. In recent decades targeted delivery of anticancer drugs specifically to the tumor cells has been widely investigated [4], [5]. Although, targeted delivery using antibodies is very effective but high expense and restriction to the usage of a variety of drugs limit its applications. A common phenomenon reported frequently in literature is the over expression of the epidermal growth factor receptor [6]–[8], transferrin receptor [9] and folate receptor in many kinds of human cancers.

Among these receptors, folate receptor (FR) is a glycosylphosphatidylinositol- anchored glycoprotein, with an apparent molecular weight of 38–40 kDa [10]. Its correspondence, folic acid (Folate, FA) due to its high binding affinity for FR, has widely been used as a targeting ligand to deliver therapeutic agents to cancer cells. Several other molecules are also reported in literature for targeted delivery [7], [11]–[13] but none of them has been found to be as promising as folic acid. Folic acid is a ligand with high affinity for the folate receptors and is very useful in targeting cell membrane for improving nanoparticle endocytosis. As a ligand folic acid has several advantages like high stability, low molecular weight, ease of accessibility and high affinity to folate receptors [14], [15]. In addition, poor immunogenicity and high stability towards organic solvents makes it an attractive option for further organic synthesis and modifications. The folate receptor α (FRα) is over expressed on many human epithelial cancer cell surfaces including cancers of breast, ovary, uterus, colon and lung [16], [17]. Thus, the therapeutic efficiency of folic acid as a ligand for folate receptors lies in their high expression levels in these types of malignancies than other normal cells.

Folate-conjugated liposomes are already reported to show enhanced cellular uptake and antitumor efficacy [18]–[20]. But one of the most recent trends of folate targeting in the literature focuses on attaching folic acid to polymer micelles [21]–[23]. Polymeric micelles are made of amphiphilic copolymers having a hydrophobic as well as a hydrophilic end. These polymer micelles are in nanometer range which not only helps in escaping the renal exclusion and reticuloendothelial system elimination but also gives them an enhanced vascular permeability. The attachment of folate to the polymer micelles further enhances their ability of recognizing tumor cells.

The purpose of this study was to compare polymer micelles with and without folate for cellular uptake and cytotoxicity on FR-positive breast cancer cell line, MDAMB231. In this study, poly(δ-valerolactone)/poly(ethylene glycol)/poly(δ-valerolactone)-folate (FVEV) was synthesized and characterized to form micelles for encapsulating anticancer drug, doxorubicin (DOX). The anticancer ability of DOX loaded poly(δ-valerolactone)/poly(ethylene glycol)/poly(δ-valerolactone)-folate micelles (FVEVDMs) was studied in MDAMB231 cell line by evaluating the cellular uptake, cytotoxicity, apoptosis and cell cycle perturbations in comparison to free DOX and DOX loaded (δ-valerolactone)/poly(ethylene glycol)/poly(δ-valerolactone) micelles (VEVDMs) without folate.

## Materials and Methods

δ-valerolactone (VL), Folic acid, doxorubicin hydrochloride (DOX), Dicyclohexyl carbodiimide (DCC), N-hydroxysuccinimide (NHS), 1-Ethyl-3-(3-dimethylaminopropyl) carbodiimide (EDC), 4-dimethylaminopyridine (DMAP), stannous octoate, 3-(4, 5-dimethylthiazol-2-yl)-2, 5-diphenyltetrazolium bromide (MTT), Pluronic F-68, Ribonuclease A, 1,6-diphenyl-1,3,5-hexatriene (DPH) and Annexin V apoptosis detection kit were purchased from Sigma-Aldrich, Steinheim, Germany. Polyethylene Glycol 2000 (PEG) was obtained from Merck Schuchardt OHG, Germany. Poly (ADP-ribose) polymerase (PARP) was bought from cell signaling and enhanced chemiluminescence kit from GE Amersham, New Jersey, USA. Human breast adenocarcinoma (MDAMB231) cells were provided from ATCC (USA) and maintained in DMEM medium containing 10% fetal bovine serum (Sigma, USA) and 1% antibiotic antimycotic cocktail (Himedia, India).

### Preparation of folic acid conjugated poly(δ-valerolactone)/poly(ethylene glycol)/poly(δ-valerolactone) (VEV-FOL)

The synthesis of triblock copolymer VEV was done by ring opening polymerization of PEG and VL in the presence of stannous octoate as catalyst and characterized by IR and NMR, as reported [24]. The synthesis of folate conjugate VEV (VEV-FOL) was done by a novel method that involves two-step reaction: Folic acid (FA) activation and its conjugation to VEV to form VEV-FOL.

### Folic acid activation

1 g of FA was activated by DCC and NHS in 5 mL DCM (molar ratio of FA:DCC:NHS = 1∶1.1∶1.1) at room temperature under nitrogen gas for 24 h. The resultant solution was filtered to remove the by-product dicyclohexylurea. Activated FA was precipitated using ice-cold ether and dried under vacuum.

### Esterification

The hydroxyl group of VEV was conjugated to the carboxylic group of FA through the ester bond. Activated FA (0.02 mmol) and VEV (0.02 mmol) were dissolved in DMF. To this, EDC (0.02 mmol) and DMAP (0.02 mmol) were added. After 24 h, the reaction solution was evaporated and dialyzed in distilled water using a dialysis tube (3,500 MW) for 48 h. Finally, the folic acid conjugated poly(δ-valerolactone)/poly(ethylene glycol)/poly(δ-valerolactone) (VEV-FOL) was obtained on freeze drying.

### Characterization of poly(δ-valerolactone)/poly(ethylene glycol)/poly(δ-valerolactone)-Folate

The chemical structure of synthesized VEV-FOL conjugate copolymer was characterized by FT-IR spectrometer (Nicolet 5700) using potassium bromide (KBr) pellets. Proton nuclear magnetic resonance spectrum (^1^H NMR) was obtained on Bruker 500 MHz using deuterated chloroform (CDCl_3_) as solvent.

### Preparation and characterization of DOX loaded poly(δ-valerolactone)/poly(ethylene glycol)/poly(δ-valerolactone)-Folate micelles (FVEVDMs)

Doxorubicin loaded VEV-FOL micelles (FVEVDMs) were prepared by solvent evaporation method. DOX (1∶100 w/w) dissolved in methanol (1∶10 v/v) was added to VEV-FOL (100 mg) solution in acetone to form the organic phase, which on addition to an aqueous phase containing Pluronic F-68 (1%) gave emulsion containing micelles. Sonication and solvent evaporation followed by centrifugation and lyophilization gave FVEVDMs in dry powder form.

Size analysis of FVEVDMs was done using particle size analyzer (Beckman Coulter Delsa Nano Particle Analyzer) and imaged using a transmission electron microscope (TEM, JEOL 1011, Japan).

Weighed amount of dried drug loaded micelles were dissolved in DMSO (dimethyl sulphoxide) and the DOX content in micelles was calculated according to a standard curve obtained using DMSO solutions of known concentrations of free DOX by UV spectrophotometer (Perkin Elmer, USA) at the detection wavelength 480 nm. The encapsulation efficiency was expressed as the ratio of DOX entrapped in micelles to the initial amount of drug used in formulation. The yield corresponds to the ratio of amount of micelles recovered to the total amount of VEV-FOL and DOX used.

To analyze the release profile of doxorubicin from micelles, DOX loaded micelles with (FVEVDMs) and without folate (VEVDMs) were dispersed in distilled water (1 mg/ml), placed in a dialysis bag (M_W_ cut off: 3500) and immersed in 15 ml of buffer solutions having pH 7.4 and incubated at 37°C. At specific time intervals, the drug released solution was replaced with equal amount of fresh media and the amount of DOX released was evaluated using UV spectrophotometer at 480 nm.

### Cell culture

To evaluate the variation in the anticancer activity of the micelles, cellular uptake and cytotoxicity experiments were conducted in breast adenocarcinoma, MDAMB231 cell line. MDAMB231 cells were cultured in DMEM medium supplemented with 10% fetal bovine serum at 37°C and 5% carbon dioxide in a humidified atmosphere. On 70% confluence, the cells were trypsinized with buffered saline solution containing 0.25% trypsin and 0.03% EDTA, and were plated to culture plate as desired. After 24 h, different concentrations of micellar formulations in cell culture medium were immediately added to the cells and analyzed as desired.

### Cellular uptake studies

To visualize the intracellular fluorescence of DOX on cellular uptake, cells were grown on cover slips placed in 24 well plates, treated with 1 µM DOX formulations and incubated for 2 h. The cells were washed, mounted and examined under a confocal laser scanning microscope (CLSM, Leica DMI 4000B) at a magnification of 60× for intracellular DOX fluorescence. The intensity of DOX fluorescence on cellular uptake was compared using histograms by flow cytometry (FACS Aria, BD, USA). Cells seeded in six-well culture plates (5×10^4^) after 24 h incubation, were treated with 1 µM DOX formulations for 2 h. The cells were then washed thrice with cold PBS, harvested using trypsin-EDTA and analyzed for DOX fluorescence using flow cytometer.

### MTT assay

To measure and compare the cytotoxicity of micelles, MTT assay was conducted. Briefly, post 24 h seeding, MDAMB231 cells (5.0×10^3^/well) were incubated with DOX micelles with and without folate at different concentrations for 24 and 72 h. Following the treatment amount of formazan crystals formed was measured after 4 h of MTT addition (10% v/v) by adding isopropyl alcohol and OD measurement at 570 nm. The relative cell viability in percentage was calculated as (A_test_/A_control_)×100. Blank micelles (100 µM) were also checked for their toxicity after incubation of 72 h.

### Annexin V-PI staining

Apoptosis was evaluated using Annexin V-FITC apoptosis detection kit by flow cytometry analysis using Annexin V-FITC staining with and without propidium iodide (PI) [25]. To measure annexin shift, MDAMB231 cells treated with or without drug (0.01 µM) for 24 h were washed in cold PBS and resuspended in binding buffer. Cells were stained with 3 µl FITC-labeled annexin and analyzed by flow cytometry. For double staining, 5 µl of PI was added 5 minutes prior to flow cytometric analysis carried out using FACS Aria (Special order system, BD, USA).

### Western blot analysis

Cleavage of the DNA repairing protein, PARP was compared using western blot analysis. MDAMB231 (2×10^6^) cells treated with 2 µM DOX formulations for 24 h were lysed and the total protein content was measured using Bradford's reagent. 50 µg of total protein was loaded for SDS-PAGE and immunoblotting carried out using antibodies specific for PARP was detected using enhanced chemiluminescence (ECL) kit [25].

### Cell cycle analysis

For cell cycle analysis, 10^6^ cells were seeded in six-well culture plates and treated with 0.01 µM of drug formulations for 24 h. Cells were harvested, fixed with 70% ethanol for 1 h and then given RNAse A (100 mg/ml) treatment for 1 h at 37°C. Propidium iodide (10 mg/ml) was added and incubated for 15 min just before the cells were analyzed using FACS Aria (Special order system, BD, USA) [25].

### Statistics

All the measurements were done in three or more replicates and the results are expressed as mean ± standard error on the mean (S.E.M). Statistical difference and level of significance (*p<0.05, **p<0.01, ***p<0.001) were calculated using the software GraphPad Instat 3.

## Results

### Synthesis and characterization of poly(δ-valerolactone)/poly(ethylene glycol)/poly(δ-valerolactone)-Folate (VEV-FOL)

**Figure 1:**
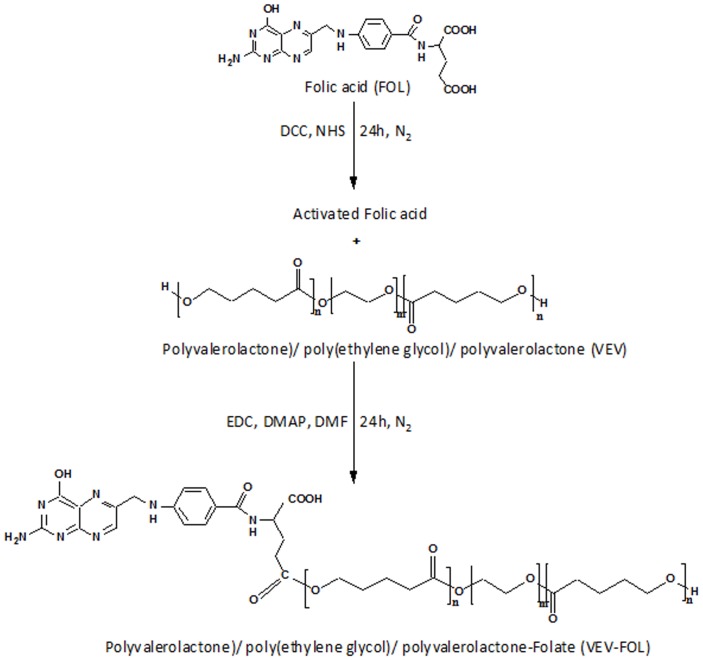
Schematic representation for the synthesis of Poly(δ-valerolactone)/Poly(ethylene glycol)/Poly(δ-valerolactone)-Folate (VEV-FOL) by folic acid activation followed by esterification.

The detailed procedure for successful synthesis of VEV has been described [24]. In brief, triblock amphiphilic copolymer of δ-valerolactone and PEG_2000_ was synthesized by ring opening polymerization technique using stannous octoate and its chemical structure was confirmed using FT-IR and ^1^H NMR. The reaction scheme for the modification of VEV by conjugation with folic acid to synthesize VEV-FOL is shown in Figure 1. In a novel procedure, the activation of –COOH group of folic acid in the presence of DCC and NHS was followed by esterification with the –OH group of VEV in the presence of EDC and DMAP as catalysts.

**Figure 2:**
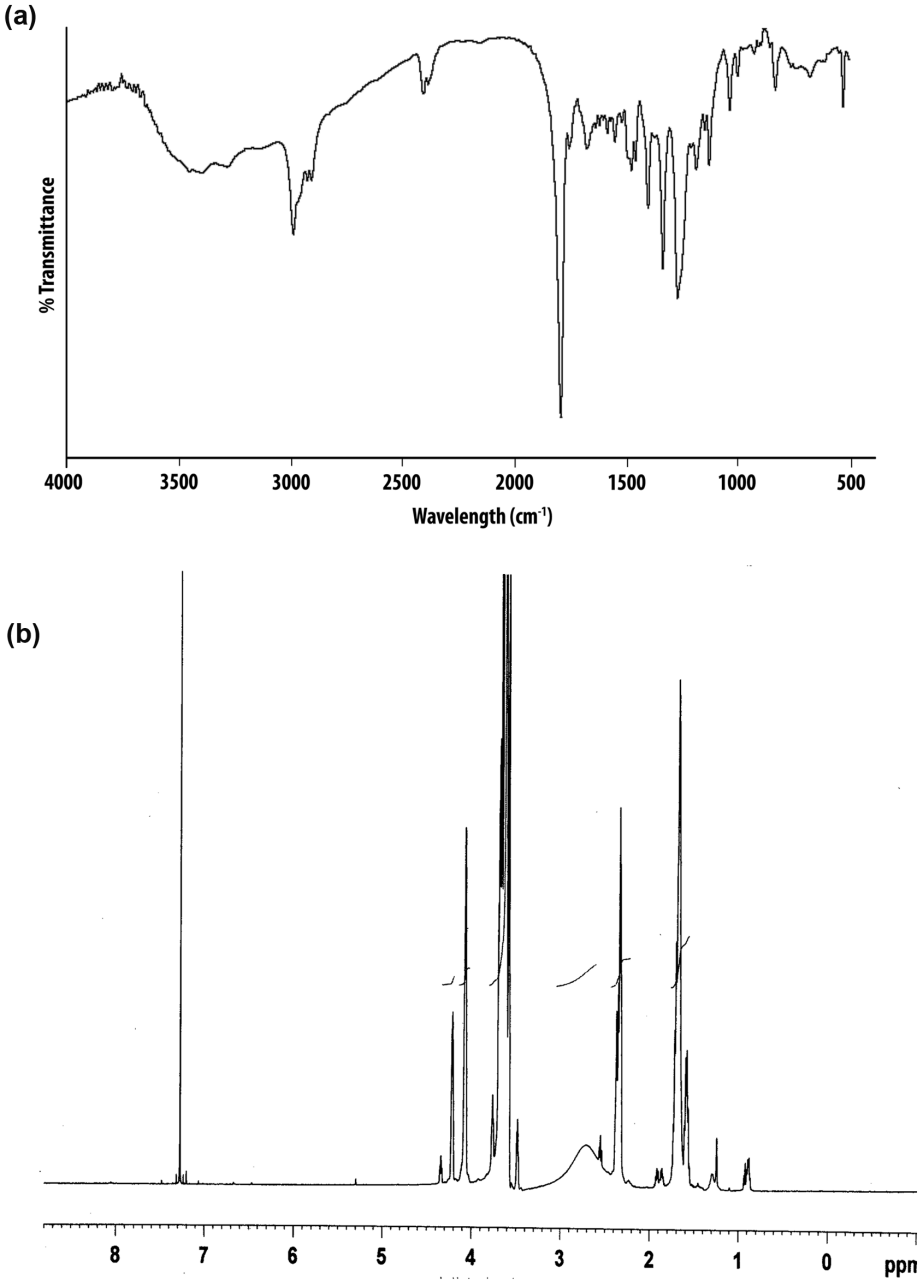
Characterization of Poly(δ-valerolactone)/Poly(ethylene glycol)/Poly(δ-valerolactone)-Folate (VEV-FOL) by (a) Fourier Transform Infra Red spectra (FT-IR) recorded using potassium bromide pellets and (b) ^1^H Nuclear Magnetic Resonance spectra (^1^HNMR) recorded in CDCl_3_ as solvent.

The chemical structure of the modified copolymer VEV-FOL was confirmed using FT-IR and ^1^H NMR spectroscopy. In FT-IR spectra, the characteristic bands at 1470 cm^−1^ and 1605 cm^−1^ represent the stretching in backbone of aromatic ring of folic acid. The band at 1514 cm^−1^ and 2958 cm^−1^ correspond to NH of –CONH and C-H stretching of aromatic ring of folate, respectively (Figure 2a).

The ^1^H NMR spectra acquired in deuterated chloroform contained signals from the protons of PEG, PVL and Folic acid. The chemical shift at ∼3.6 ppm corresponds to PEG and the chemical shifts at 2.4 ppm, 1.6 ppm and 4 ppm are of δ- valerolactone. The small peaks at 1.8, 6.6, 7.1 and 8.1 ppm are the typical protons of folate (Figure 2b), confirming the successful synthesis of VEV-FOL copolymer.

### Characterization of DOX loaded VEV-FOL micelles (FVEVDMs)

**Figure 3:**
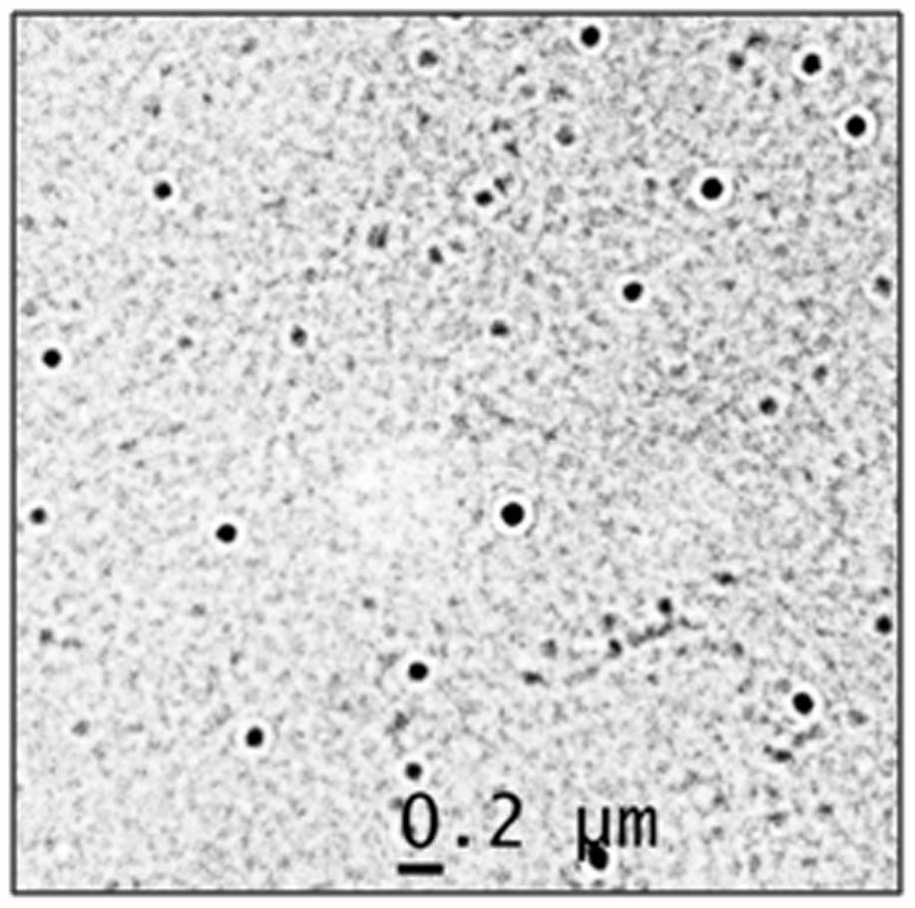
Transmission Electron Microscope (TEM) image of doxorubicin loaded Folate conjugated VEV micelles (FVEVDMs).

A novel single emulsion method was employed to prepare DOX loaded VEV-FOL micelles having an inner PVL core with the surface decorated with folate and PEG. These micelles showed a mean diameter of 97 nm (Figure 3) with encapsulation efficiency of 62% and polydispersity (0.19±0.02) (Table 1). Interestingly, no increase in size of the micelles was observed on modifying the VEV copolymer with folic acid.

**Table 1:** Characterization of doxorubicin entrapped Poly(δ-valerolactone)/Poly(ethylene glycol)/Poly(δ-valerolactone)-Folate micelles (FVEVDMs).

Sample	Encapsulation efficiency %	Diameter (nm)	Yield %	Polydispersity
FVEVDMs	62±2.4	94±4	85±3.7	0.19±0.02

(Figure 4) shows the release profile of DOX from copolymeric micelles with and without folate. The folate conjugated micelles (FVEVDMs) showed a biphasic pattern characterized by an initial burst of 40% in first 24 h followed by a slow and sustained release. The initial burst may be attributed to the DOX molecules present at the surface. FVEVDMs were able to sustain DOX release for more than two weeks and showed a similar pattern as that of micelles without folate (VEVDMs).

**Figure 4:**
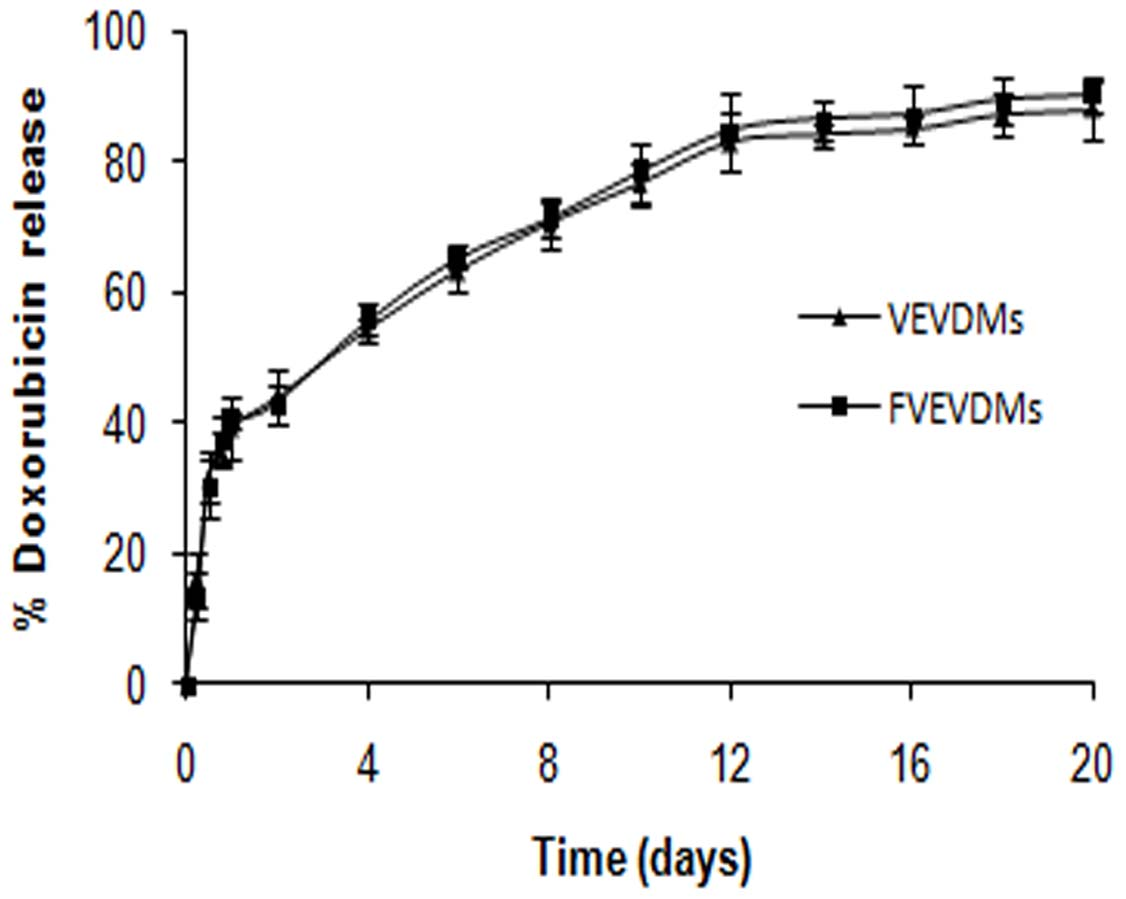
*In vitro* release of doxorubicin from FVEVDMs and VEVDMs: Release pattern of DOX from micelles with and without folate in phosphate buffer at pH 7.4 and 37°C.

### Folate conjugation enhanced cellular uptake significantly

Our previous results have already shown that the VEVDMs showed almost 2–3 folds increase in cellular uptake in comparison to free DOX. To analyze the changes in cellular uptake on conjugation to folic acid, intracellular fluorescence of DOX was analyzed in FR-positive MDAMB231 cell line using confocal microscopy and the comparison of fluorescence intensity was done using flow cytometer.

**Figure 5:**
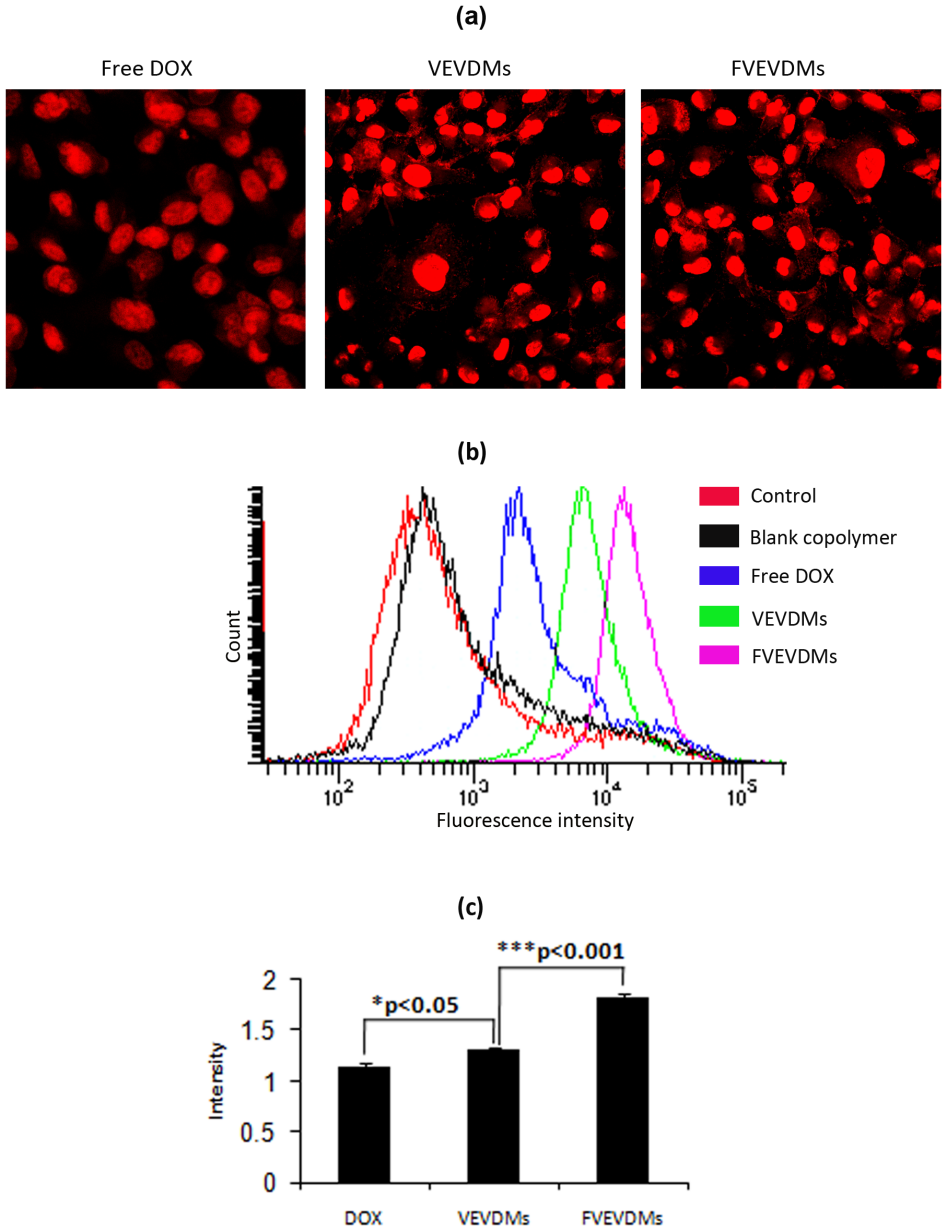
Cellular uptake showing comparison of intracellular fluorescence of doxorubicin: Incubation of MDAMB231 cells with different doxorubicin formulations (1 µM) for 2 h is shown by (a) Confocal microscopy images showing intracellular fluorescence of doxorubicin (b) Flow cytometry histograms showing intensity of uptake (c) Comparison of uptake efficiency by intensity of doxorubicin fluorescence. Bars marked with *p<0.05,***p<0.001 shows the level of significance of difference at the same concentration (n = 3).

Confocal cell images were used to determine intracellular doxorubicin localization and accumulation in the cells incubated with free DOX, VEVDMs and FVEVDMs (Figure 5a). The comparison of fluorescence intensity using flow cytometer showed further enhancement in uptake by micelles with folate in comparison to micelles without folate (Figure 5b). DOX loaded micelles having folic acid on their surface showed significant increment in uptake efficiency of doxorubicin (Figure 5c) showing that folate conjugated micelles have better selectivity over FR expressing cells.

### DOX loaded VEV-FOL micelles exhibited significantly better cytotoxicity

**Figure 6:**
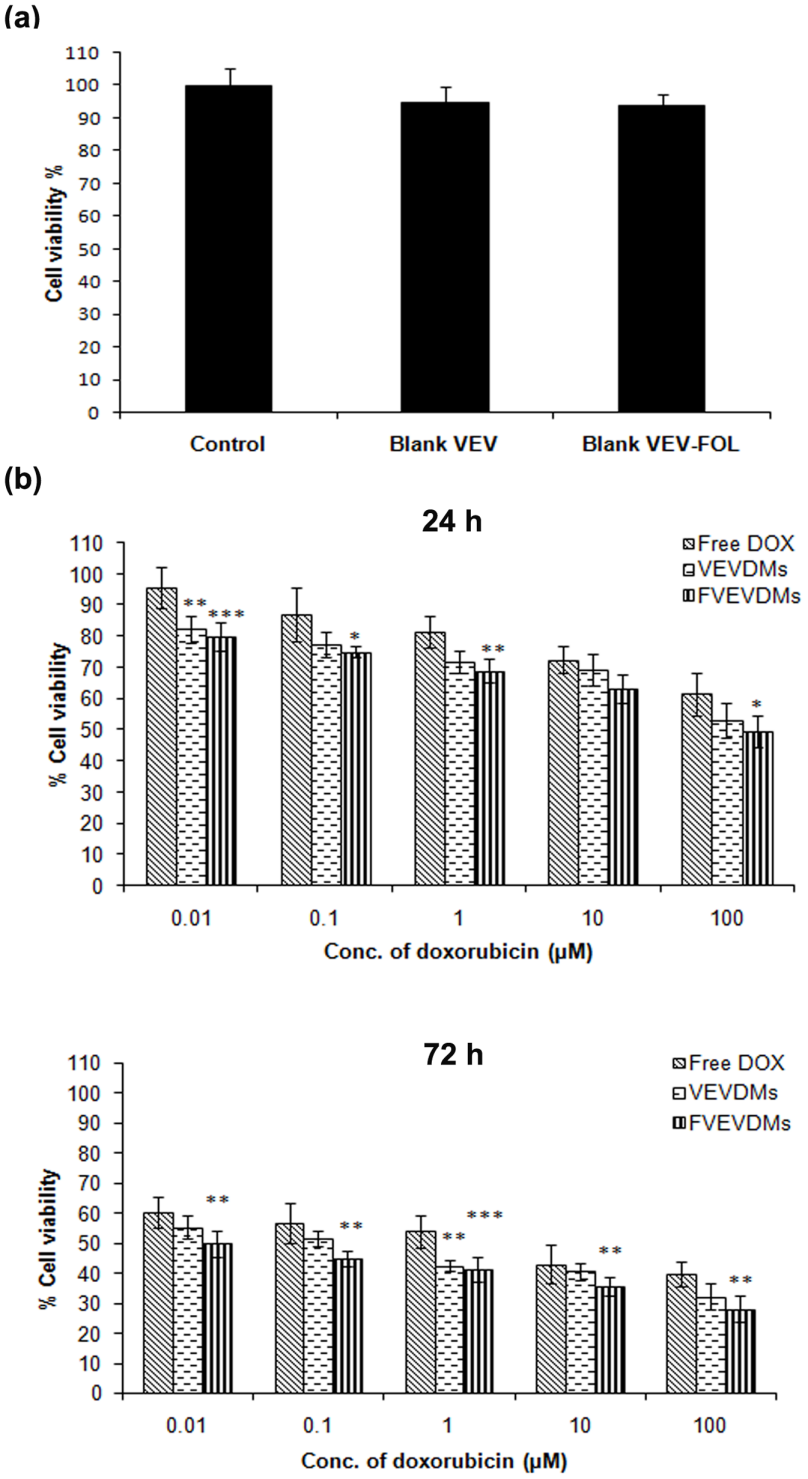
MTT assay. Cytotoxicity to MDAMB231 cells induced by (a) Blank micelles (100 µM) on incubation for 72 h (b) Different DOX formulations at different concentrations and time durations, as indicated. All the measurements were done in six replicates and the results are expressed as arithmetic mean ± standard error on the mean (S.E.M). Bars marked with *p<0.05,**p<0.01,***p<0.001 shows the level of significance of difference from free drug at the same concentration.

Without DOX loading, no toxicity was observed after 72 h of incubation of 100 µM VEV micelles, with and without folate (Figure 6a). The cytotoxicity of different DOX formulations (free DOX, DOX loaded VEV and VEV-FOL micelles) to MDAMB231 cells were compared. With equivalent drug concentrations in the culture medium, the VEV-FOL micelles (FVEVDMs) showed much higher cytotoxicity than VEV micelles and free DOX at 0.01–100 µM drug concentrations for incubation of 24 and 72 h (Figure 6b). FVEVDMs exhibited enhanced cytotoxicity with almost ten times smaller IC_50_ value of 0.014 µM when compared to 1.39 and 0.11 µM for pristine DOX and VEV micelles without folic acid, which may be attributed to their improved cell uptake.

### Apoptosis assays prove the superior anticancer activity of folate conjugated micelles

**Figure 7:**
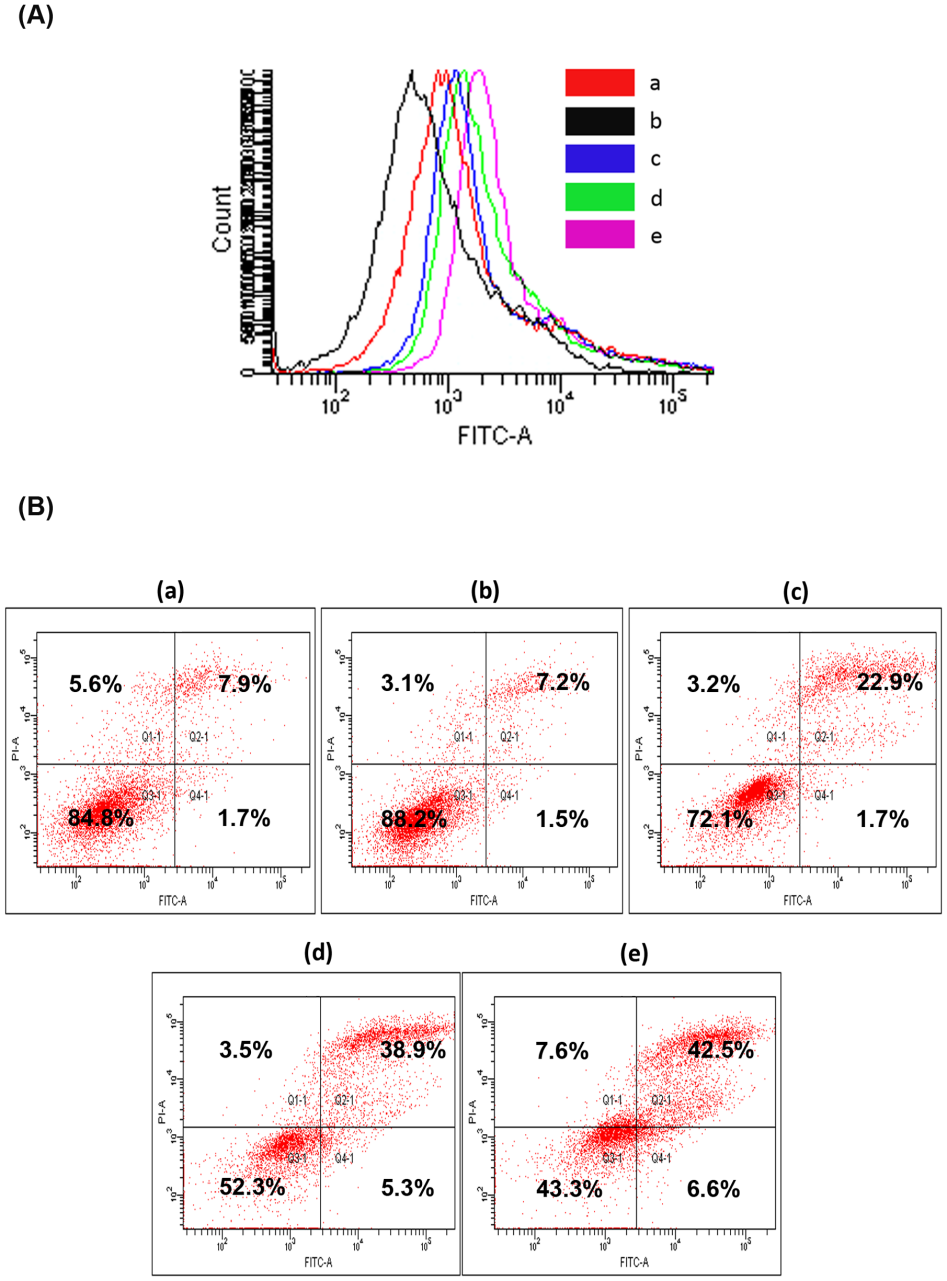
Annexin V-PI stain assay. (A) Apoptosis determined by flow cytometry in MDAMB231 cells treated with different DOX fomulations (0.01 µM) for 24 h after staining with Annexin V-FITC. Cells showing higher fluorescence (shift towards right) represent apoptosis (B) FACS analysis of Annexin V and PI in MDAMB231 cells treated with different DOX formulations (0.01 µM) for 24 h where Annexin V+ PI+; Annexin V+ PI−; Annexin V− PI+ and Annexin V− PI− indicate late apoptotic; early apoptotic; necrotic and live cells where a–e represent treatments as a:Control; b:Blank copolymer; c:Free DOX; d:VEVDMs; e:FVEVDMs.

Apoptosis induced by different DOX formulations at a concentration of 0.01 µM for 24 h was analyzed using Annexin V-FITC staining. It identifies cell surface changes that occur in the early stages of apoptosis and show a shift towards increasing fluorescence in the FACS histogram due to fluorescence emitted by apoptotic cells. Flow cytometry data suggested that approximately 41% cells showed positive annexin staining after treatment with folate conjugated micelles. However, only 29% and 18% of MDAMB231 cells were positive after treatment with DOX loaded micelles without folate and free DOX (Figure 7A).

To further analyze the features of cell death, double staining with Annexin V-FITC and PI was done and the percentage of annexin V + and PI + MDAMB231 cells was evaluated by two-fluorescence flow cytometric analysis. Only 26% and 42% cells were annexin V + PI + (late apoptotic/necrotic) on treatment with 0.01 µM of free DOX and VEVDMs. Also, 1.7% and 5.3% cells were annexin V + PI− (early apoptotic) with these treatments. However, treatment with FVEVDMs showed 50% annexin V + PI + (late apoptotic/necrotic) cells and 6.6% annexin V + PI− (early apoptotic) cells (Figure 7B). These results indicate the efficiency of folate conjugated micelles to induce better apoptosis than other treatments.

**Figure 8:**
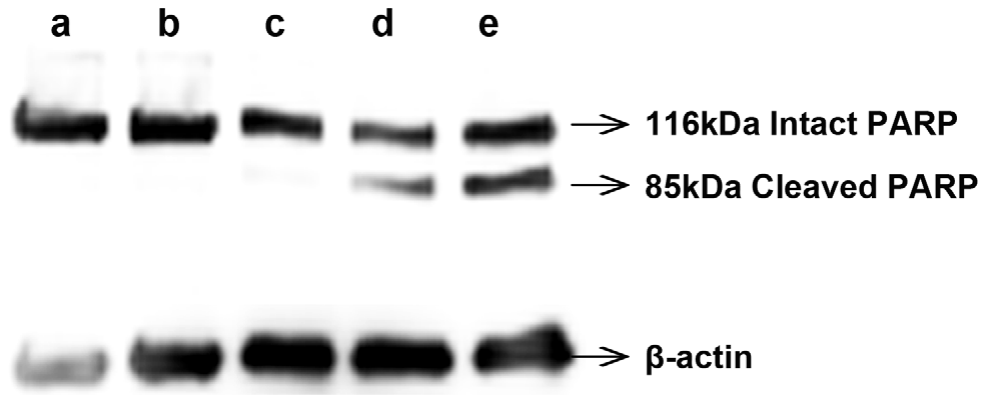
Western blot analysis. Comparison of PARP cleavage induced in MDAMB231 cells treated with different DOX formulations (2 µM) for 24 h where a–e represent a: Control; b: Blank copolymer; c: Free DOX; d: VEVDMs; e: FVEVDMs. Immunoblotting was carried out using antibodies specific for PARP and detected using enhanced chemiluminescence method.

To support these data, PARP cleavage, a clear indicator of apoptosis was analyzed using western blot. Immunoblot results showed that incubation with 2 µM of DOX formulations induced PARP cleavage with clearly much higher intensity of the 85-kDa cleaved PARP in sample incubated with folate micelles (Figure 8e) whereas free DOX showed negligible cleavage (Figure 8c), indicating the superior antitumor efficacy of FVEVDMs.

### Better G2/M arrest induced by FVEVDMs

**Figure 9:**
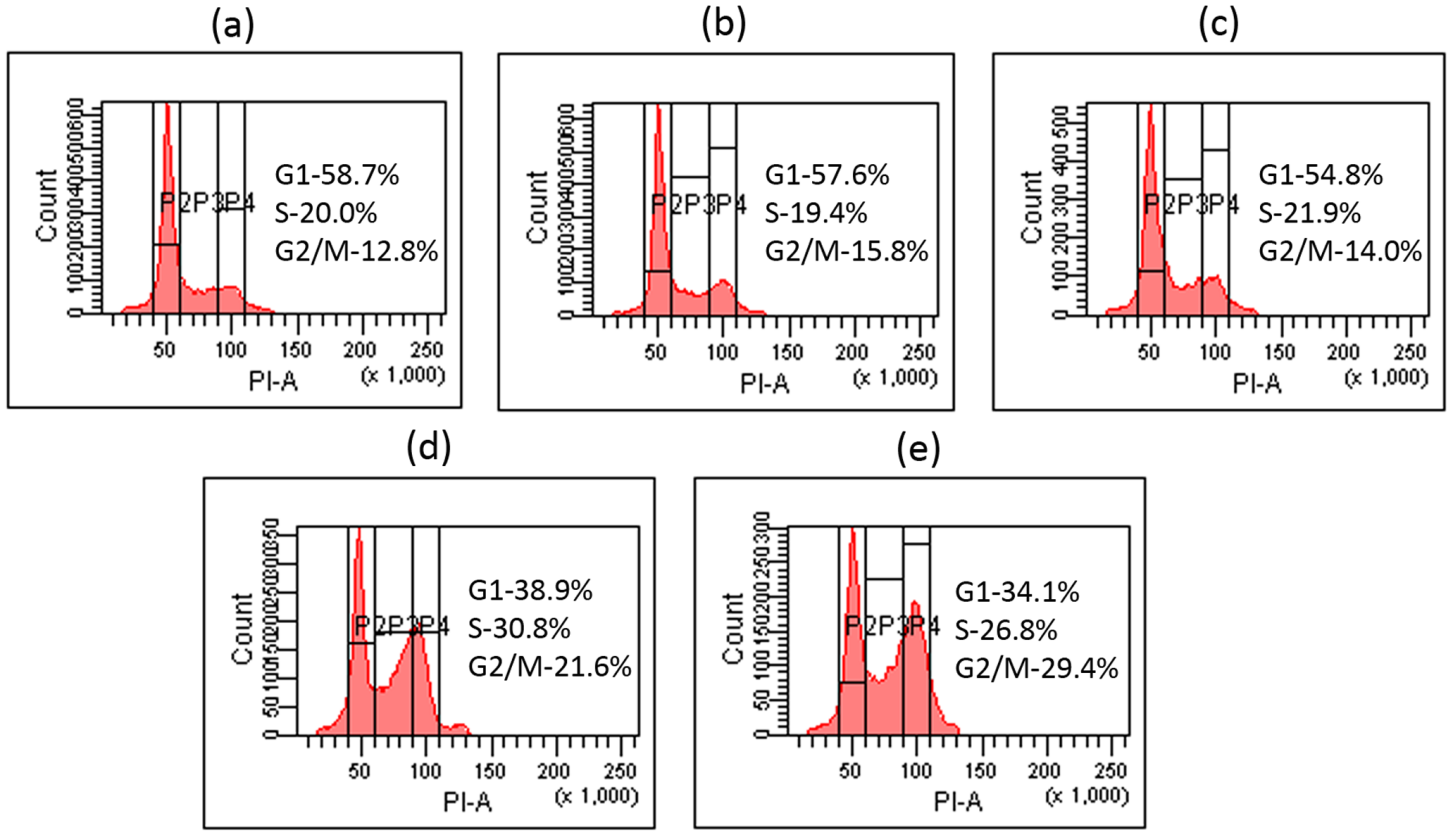
Cell cycle analysis. Variation in cell cycle of MDAMB231 cells on treatment with different DOX formulations (0.01 µM) for 24 h analyzed using FACS.

Since doxorubicin is reported to induce G2/M phase arrest, the cell cycle perturbations on treatment with different DOX formulations was analyzed using flow cytometer. At a very low concentration of 0.01 µM and incubation time of 24 h, free DOX did not show any activity. However, doxorubicin micelles showed a significant G2/M arrest with folate conjugated micelles arresting the highest and almost double percentage of MDAMB231 cells (30%) when compared to free drug (Figure 9).

## Discussion

One of the major challenges in chemotherapy is non-specific delivery of anticancer agents to healthy tissues leading to various side effects. There have been numerous studies for the development of site specific delivery agents [26], [27], among which folic acid based nanotechnology has come up with very promising results [28]. Folic acid is a stable and inexpensive chemical which can be covalently conjugated to different molecules. Moreover, the ability of folic-acid conjugated molecules to be taken up by the cancer cells that overexpress folate receptors makes it a potential ligand for targeted drug delivery [10].

In this study, poly(δ-valerolactone)/poly(ethylene glycol)/poly(δ-valerolactone) copolymer was modified with folic acid conjugation (VEV-FOL), characterized and its antitumor efficacy on doxorubicin encapsulation (FVEVDMs) in comparison to free DOX and DOX loaded VEV micelles without folate (VEVDMs) was evaluated in FR-positive breast cancer cell line, MDAMB231. The synthesis of VEV-FOL was done successfully by a novel procedure that includes folic acid activation [22] followed by esterification [29] (Figure 1). The FT-IR and ^1^H NMR spectra of VEV-FOL (Figure 2a & 2b) confirmed the successful conjugation of folic acid to VEV [22], [30]. However, the weaker intensity of folate peaks in the ^1^H NMR spectra is probably due to small molecular weight of folate in comparison to VEV. In agreement to previous reports, conjugation of folic acid to VEV copolymer did not alter the size, encapsulation efficiency and polydispersity of doxorubicin micelles (Table 1). Particle size is an important factor that directly affects the cellular uptake and biodistribution from the nanocarriers. In our study FVEVDMs obtained in small size (97 nm) (Figure 3) may help in escaping the reticuloendothelial system which prevents their uptake by macrophages. These micelles showed a slow and sustained release kinetics (Figure 4) [23] which helps in avoiding the frequent administration of drug.

**Figure 10:**
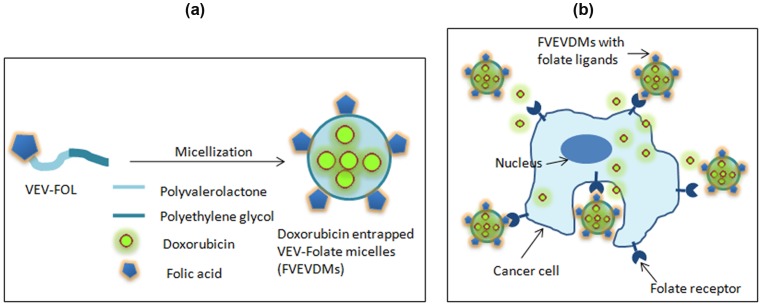
Schematic representation of the structure of FVEVDMs and their targeting ability. (a) Diagram showing the structure of DOX entrapped VEV-FOL copolymeric micelles having hydrophobic PVL core containing drug surrounded by PEG and folic acid as targeting molecules on the surface (b) Active cellular targeting ability of VEV-FOL micelles by specific binding to target cells and drug release on cellular internalization or in their close proximity.

Presence of molecules like folic acid helps in achieving active cellular targeting by cell-specific recognition and binding [28], [31]. Doxorubicin entrapped VEV-FOL micelles (FVEVDMs) due to its amphiphilic nature have stable polyvalerolactone core surrounded by PEG blocks and the targeting molecule, folic acid (Figure 10a). Since, breast cancers are reported to over express folate receptors, MDAMB231 cell line was selected [32] to analyze the impact of folic acid conjugation on cellular uptake and antitumor efficacy of DOX entrapped micelles with respect to pristine DOX. These micelles are expected to release the drug specifically into the targeted cells or their close proximity, making them potential for targeted drug delivery (Figure 10b).

Due to natural fluorescence of doxorubicin, DOX formulations (1 µM) were directly used to measure cell uptake without additional markers using confocal microscopy. From the red fluorescence of DOX, its distribution in the cells can be clearly observed (Figure 5a). The drug was seen mainly concentrated in the nuclei, which is a characteristic of DOX whereas the micelles showed slight fluorescence in the cytoplasm also, indicating their internalization by endocytosis [33]. Since the fluorescence intensity is proportional to the amount of drug internalized by the cells, better intensity of DOX micelles is indicative of their better cellular uptake [19], [22]. The intensity of DOX fluorescence measured using flow cytometry with equivalent DOX concentration in each formulation and time showed highest intensity with FVEVDMs than free DOX and VEVDMs (Figure 5b). Quantification of uptake clearly indicated that the cellular uptake of micelles can be enhanced significantly by attachment of folate on their surface (Figure 5c).

Conjugation of folic acid to the micelles did not impart any toxicity to MDAMB231 cells. However, DOX encapsulated micelles conjugated with folic acid (FVEVDMs) exhibited significantly better cytotoxicity, which became more pronounced over a longer period (Figure 6). This is evident from the 10 fold decrease in the IC_50_ value of micelles after conjugation of folic acid. It can be considered as a consequence of receptor mediated endocytosis of folate micelles by binding to FRs which results in enhanced cellular uptake, in comparison to the other two treatments [16], [21]. These results support our hypothesis that conjugation of a targeting moiety like folic acid to VEV micelles further enhances its therapeutic efficiency in comparison to unconjugated micelles and native drug.

Analysis of apoptotic nature of these DOX formulations was done by Annexin V-FITC and PI staining and PARP cleavage. Apoptosis is a phenomenon accompanied by change in plasma membrane structure by surface exposure of phosphatidylserine (PS) without losing the membrane integrity. Based on PS exposure to outer leaflet and affinity of Annexin V-FITC to bind to PS, Annexin V-FITC assay was described to detect apoptosis [34]. Annexin V binds to dead cells which loses their integrity but is not able to bind to live cells since the molecule is not able to penetrate their phospholipid bilayer. Moreover, necrotic and apoptotic cells are discriminated by adding the membrane impermeable DNA stain, propidium iodide (PI). Thus, not only from the shifting of the peak of Annexin V-FITC towards right, but also from dual staining with PI, the cells undergone apoptosis were measured (Figure 7). Higher percentage of Annexin V (+ve) and PI (+ve) cells clearly indicated the better efficiency of folate micelles (FVEVDMs) to cause cell death in a programmed way, rather than causing necrosis [35]. During apoptosis, PARP- a 116 kDa nuclear protein that is normally involved in a number of cellular processes, mainly DNA repair and programmed cell death, is cleaved to yield p85 and p25 fragments [36]. Sensitive detection of PARP cleavage therefore easily allows the detection of apoptosis. The higher intensity of the 85 kDa cleaved PARP band on treatment with equal concentration (2 µM) of FVEVDMs (Figure 8), a concentration at which free DOX almost failed to cause any PARP cleavage, clearly shows the superiority of folate VEV micelles over other treatments.

Doxorubicin, which is known to induce DNA damage predominantly in the G2/M phase of cell cycle [37], failed to show any activity at a low concentration of 0.01 µM. However, a significant G2/M arrest was induced by the same concentration of folate mediated micelles which was higher than that of micelles without folic acid (Figure 9). Since doxorubicin induced cell cycle arrest is concentration and exposure time dependent [38], [39], low concentrations of FVEVDMs may serve the same purpose as done by higher dose of pristine doxorubicin. This might be due to higher concentration of drug available at the site of action for a longer period than native drug in solution which is a consequence of higher cellular uptake and sustained release. Although, further studies to analyze the *in vivo* behavior of FVEVDMs are required, our results clearly indicate that with the presence of PEG and folic acid, VEV-FOL can be a competent carrier for effective targeted chemotherapy.

## Conclusions

A novel folic acid mediated δ-valerolactone and PEG based copolymeric micellar system was successfully synthesized and characterized for targeted delivery of doxorubicin. DOX loaded VEV-FOL micelles were obtained with a mean diameter of 97 nm and showed sustained release that continued for more than two weeks. The significant improvement in cellular uptake, cytotoxicity and efficiency to induce apoptosis by FVEVDMs in comparison to free DOX and VEVDMs clearly indicate that these novel folate conjugated copolymeric micelles have strong potential against cancer cells for targeted chemotherapy.
